# Enhanced efficacy of IL-15-based ALT-803 superagonist complex in combination with immune checkpoint inhibitors in hematologic and metastatic mouse tumor models

**DOI:** 10.1186/2051-1426-2-S3-P121

**Published:** 2014-11-06

**Authors:** Wenxin Xu, Warren D Marcus, Peter R Rhode, Hing C Wong

**Affiliations:** 1 Altor BioScience Corp., Miramar, FL, USA

## 

Recent advances in achieving highly durable clinical responses via inhibition of immune checkpoint molecules have revolutionized the outlook for cancer immunotherapy. However, such responses are only observed in a minority of patients, suggesting that strategies to further augment antitumor immune activity may provide additional clinical benefit. ALT-803 is an IL-15 superagonist:IL-15Rα-Fc complex capable of stimulating T cell and NK cell responses without inducing Treg activity. ALT-803 has improved pharmacokinetics and biodistribution compared to native IL-15, allowing this complex to exhibit more potent efficacy than IL-15 in various hematologic and solid tumor models. Mechanism-of-action studies showed that ALT-803 is capable of inducing both innate effector-like and adaptive T cell antitumor activity, suggesting that the immunostimulatory effects of ALT-803 could compliment the activity of checkpoint inhibitors to block tumor immune evasion. To assess this, we utilized a murine 5T33P myeloma model in which ALT-803 was shown to be effective at decreasing tumor burden in the bone marrow and prolonging animal survival. Treatment of mice bearing established 5T33P tumors (5/group) with 5 mg/kg anti-PD-L1 mAb also prolonged survival compared to controls (P > 0.01), whereas treatment with 1.25 mg/kg anti-PD-L1 mAb or 10 mg/kg anti-CTLA-4 mAb had no significant antitumor effect (p < 0.05). Consistent with these results, 5T33P cells were found to express PD-L1 but not CD80/CD86. Treatment with sub-optimal 0.25 mg/kg anti-PD-L1 mAb or 50 µg/kg ALT-803 was ineffective at prolonging survival of 5T33P-bearing mice (median survival; PBS, 27 days; anti-PD-L1 mAb, 31 day; ALT-803, 28 days). However, combination therapy with sub-optimal anti-PD-L1 mAb plus ALT-803 extended median survival to 66 days (p < 0.05 vs. controls). These combination therapies were also evaluated in a metastatic murine CT26 colon carcinoma model that was previous used to show increased efficacy of IL-15+anti-PD-L1+anti-CTLA-4 mAb treatment. Notably, ALT-803+anti-PD-L1+anti-CTLA-4 mAb treatment provided greater survival benefit to mice with metastatic CT26 tumors than treatment with vehicle or ALT-803 alone (median survival: combined therapy, 53 days vs. PBS, 15 days; vs. ALT-803, 22.5 days; both p < 0.01) or the IL-15+anti-PD-L1+anti-CTLA-4 mAb combination (median survival: 18 days; p < 0.001). In all of these models, combination therapies of ALT-803 and anti-checkpoint Abs were well tolerated. Characterization of immune cell activity responsible for improved antitumor efficacy is underway. Overall, these results confirm that enhanced antitumor responses can be achieved by combining immune checkpoint blockers with ALT-803, warranting evaluation of these strategies in the clinical setting.

## Acknowledgements

NIH/NCI grant CA167925 (Wong).

